# The perceived present: What is it, and what is it there for?

**DOI:** 10.3758/s13423-020-01726-7

**Published:** 2020-03-30

**Authors:** Peter A. White

**Affiliations:** School of Psychology, Cardiff University, Tower Building, Park Place, Cardiff, Wales CF10 3YG UK

**Keywords:** Perceived present, Temporal discrimination, Conscious perception, Sensory memory, Perceptual processing latencies

## Abstract

It is proposed that the perceived present is not a moment in time, but an information structure comprising an integrated set of products of perceptual processing. All information in the perceived present carries an informational time marker identifying it as “present”. This marker is exclusive to information in the perceived present. There are other kinds of time markers, such as ordinality (“this stimulus occurred before that one”) and duration (“this stimulus lasted for 50 ms”). These are different from the “present” time marker and may be attached to information regardless of whether it is in the perceived present or not. It is proposed that the perceived present is a very short-term and very high-capacity holding area for perceptual information. The maximum holding time for any given piece of information is ~100 ms: This is affected by the need to balance the value of informational persistence for further processing against the problem of obsolescence of the information. The main function of the perceived present is to facilitate access by other specialized, automatic processes.

Perceptions seem to have a “now” quality: Ordinarily, we take it for granted that what we perceive is what is the case or what is happening now, as if there were zero delay in perception. It is hard to think of any example of a perception that has a “past” quality, and, indeed, “perceiving the past” seems almost like a contradiction in terms. The “nowness” of perception is a kind of illusion or, more precisely, an informational construct of perceptual processing. This paper is about that construct, which, from this point on, will be termed the “perceived present”: the location in the stream of perceptual and memorial processing in which it occurs, its temporal resolution, how the quality of “nowness” is generated, and what functions the perceived present might have. It will be argued that the perceived present is an important and neglected topic in psychology and neuroscience.

Whether or not there is a present moment in the world outside perception, and whether or not time flows on from one moment to the next, are both matters of active contention in philosophy and physics (see, e.g., Al-Khalili, 2012; Barbour, 1999; Bergson, 1910; Buonomano, 2017; Dainton, 2008; Davies, 1995; Dorato & Wittmann, 2015; Greene, 2004; Gruber & Block, 2013; Smart, 1980; D. Zimmermann, 2011). This paper does not aim to take a position on those issues. However, the perceived present, whatever else it may be, cannot be the present moment of physics, even if there is one. The temporal dimension in the space-time manifold can be represented as a series of Planck time units, each defining a single coordinate on that dimension. The duration of a Planck time unit is 10^-44^ s. To put this in context, the period of a light wave, the time it takes light to travel one wavelength, is about 10^-15^ s (’t Hooft & Vandoren, 2011), which is 29 orders of magnitude longer than the Planck time. If there is a present moment in the world, it has a time scale of one Planck time unit, just because each Planck time unit has a different coordinate on the temporal dimension. The minimum possible time scale of the perceived present cannot be anywhere near the Planck time unit. The refractory period for neurons is about 1 ms, and the upper limit for consecutive firing in a single neuron is about 800 impulses per second (Goldstein, 2014). This places a fundamental limit on local temporal resolution of signals. Probably the finest temporal resolution in human sensory systems is found in audition, where differences in arrival times of sound to the two ears can be resolved on a scale of microseconds (Grothe, 2003). For most processing, however, the functional properties of neurons imply that temporal resolution in the perceived present must be on the millisecond time scale. This implies that the perceived present is not the hypothetical objective present. Like everything else in perception, it is a construct in the brain.

The perceived present is not defined in terms of its subjective or introspective qualities, though it is difficult to talk about the perceived present without seeming to implicate issues of subjectivity, introspection, and consciousness. This paper is not concerned with the nature of consciousness, either in general or with specific reference to percepts. It is possible that much if not all of the perceived present is conscious, and it is also possible that the perceived present constitutes a respectable proportion of all that is conscious. The issue of how the perceived present is constructed, however, can be addressed without considering whether the information in question is conscious or not. Also, the paper is not concerned with perception of the flow of time (see, e.g., Gruber & Block, 2013, 2017). Distinguishing the perceived present from the perceived flow of time is not so easy, because, of course, perception moves on ceaselessly. Hopefully, this matter will become clearer as the paper unfolds.

## The perceived present: A proposal

It is proposed that the perceived present is an information structure comprising products of perceptual processing, which may include low-level and high-level products, integrated into a coherent body of information. It is a very short-term holding area for information and has very high capacity. Information is held in the perceived present for a maximum of ~100 ms, though holding time may be less than that, depending on processing priorities and, probably, decay or interference. From there it is either lost or transferred to other stores, such as sensory memory and working memory. Information in the perceived present is labelled with various kinds of time markers. All and only information in the perceived present is labelled with a time marker identifying it as “present” (as opposed to “past”); it may also be labelled with other kinds of time markers, such as ordinal temporal relations, that need not be confined to the perceived present. Any information that is time-marked as “present” is part of the perceived present for as long as it carries that label. This may include products of perceptual processing in any modality including exteroception such as vision, proprioception (Proske & Gandevia, 2012), and interoception (Craig, 2009a, b), and also products of other processes in the brain such as mental simulation (White, 2012). It is proposed that the perceived present has the features it has because they are adapted for the function of accessibility to postperceptual automatic processing.

Subsequent sections address the main features of the proposed perceived present: in order, the location of the perceived present in the stream of perceptual processing, the time marking of information as “present,” and the proposed function of the perceived present as a holding area for perceptual products. A more extensive depiction of the perceived present will be presented toward the end of the paper, in light of the ground covered along the way.

## The perceived present is an integrated, coherent body of perceptual information

Part of the proposal is that the perceived present is an integrated, coherent body of perceptual information. This means (i) that it includes semantic information, such as object individuation and identification, and (ii) that it is not to be identified with information in any postperceptual store, such as sensory memory. These two propositions will be addressed in turn.

On the first proposition, that the perceived present includes categorical and other semantic information, the counterproposal would be that the perceived present, if it exists at all, is precategorical, lacking semantic information. It is not easy to disconfirm this counterproposal because it is difficult to obtain direct evidence about the contents of the perceived present. In research on perception, participants are commonly asked to report what they perceived. One problem with this was discussed by Breitmeyer, Kropfl, and Julesz (1982), in connection with visual sensory memory, but applies equally to the perceived present. It could be argued that the products of perceptual processing, and the contents of the perceived present, are precategorical, limited to surface physical features such as shapes and colours. The task of reporting what is perceived initiates entry of precategorical perceptual products into postperceptual processing, where semantic information is accessed and incorporated, and can then be reported. That would give the impression that the semantic information was part of the products of perceptual processing when in fact it was generated in postperceptual processing. Under that hypothesis, the perceived present, as a set of perceptual products, would comprise only precategorical information, and reports are not trustworthy evidence of what was perceived.

Against that, however, is abundant evidence, including EEG and neuroimaging evidence, that, even from an early stage, activated categorical and semantic information is involved in perceptual processing, through mechanisms of reentrant or recurrent processing, and that much if not all of perceptual processing can be regarded as an interplay between incoming information and preexisting informational structures of various kinds (Bar et al., 2006; Di Lollo, 2012; Di Lollo, Enns, & Rensink, 2000; Herzog & Manassi, 2015; Hochstein & Ahissar, 2002; Kahan & Enns, 2014; Lamme, 2006; Tapia & Beck, 2014). To the extent that that is the case, semantic information is integrated into perceptual processing almost, if not entirely, throughout its course. Indeed, some studies have found evidence that early, low-level, feedforward processing does not correlate with perception, and that perception represents a stage at which reentrant processing has enabled the emergence of coherent bodies of perceptual information such as individuated perceptual objects defined by integrated feature combinations (Di Lollo, 2012; Fahrenfort, Scholte, & Lamme, 2008).

A relevant example is a study by Wyatte, Curran, and O’Reilly (2012). They considered the problem of object identification with input degraded by partial occlusion or low contrast. They showed that feedforward processing alone was insufficient to account for performance on object category identification under such conditions, and that reentrant processing involving activated categorical information was required. It is of course possible that further categorical and semantic processing occurs after perceptual processing is complete. A verbal label for the category, for example, might be activated in postperceptual processing, thereby enabling a verbal report of stimulus identity. But the results of the study by Wyatte et al. (2012), and numerous others, show object identification occurring in perceptual processing as a result of activation of categorical knowledge. Moreover, effects of reentrant processing have been reported with latencies as short as 100 ms, further supporting the contention that perception involves categorical knowledge from an early stage (e.g., Lamme & Roelfsema, 2000). The evidence concerning the occurrence and time course of reentrant processing therefore supports the hypothesis that the contents of the perceived present incorporate categorical and other semantic information.

The foregoing argument has focussed on vision, but categorical and semantic information is a feature of the perceived present in other modalities as well. For example, as Gibson (1962) noted, when we grasp a mug, the tactile/haptic percept is of an identified object, not of multiple individual and local points of contact on different fingers. This indicates integration for object identification in haptic information across spatially separate sites of stimulation and, again, semantic information in a coherent, integrated perceptual representation.

On the second proposition, the counterproposal would be that the perceived present should be identified with information in a short-duration postperceptual store, specifically sensory memory. Sensory memory is a store in which perceptual information may persist up to a maximum of ~1,000 ms, though with most decay occurring within 250 ms (Auvray, Gallace, & Spence, 2011; Bliss, Crane, Mansfield, & Townsend, 1966; Darwin, Turvey, & Crowder, 1972; Demkiw & Michaels, 1976; Haber, 1983; Jacob, Breitmeyer, & Treviño, 2013; Schill & Zetzsche, 1995; Sligte, Vandenbroucke, Scholte, & Lamme, 2010; Sperling, 1960). Could the perceived present be identified with information in sensory memory? One problem for that hypothesis is that, under laboratory experimental conditions, the decay of information in sensory memory can itself be reported. Anecdotally, Sperling (1960) noted that, on brief presentation of complex visual stimuli, “observers enigmatically insist that they have seen more than they can remember afterwards, that is, report afterwards” (p. 1). The veridicality of that insistence is not the issue here. The issue is that the decayed information was located by the observers in the past, not the present. That is, it is identified by the observers as the remembered (or forgotten) past, not the perceived present. Sensory memory is exactly what it seems to be, a store of information about the recent past, not the perceived present. More argument against the hypothesis that the perceived present is a feature of or can be identified with sensory memory will be presented in the section titled “Balancing Persistence Against Obsolescence”.

In conclusion to this section, then, it is likely that the hypothesized perceived present is a set of information constructed in perceptual processing, incorporating semantic information, such as object individuation and identification, and is not to be identified with information in sensory memory.

## Marking information as “present”

A central claim in the present proposal is that perceptual information is labelled or marked with different kinds of temporal information, and that the distinguishing feature of the perceived present is that all and only information in it is marked as “present”. This marker is inaccurate because the information that enters the perceived present is running some way behind the stimuli that gave rise to it, a matter that will be further discussed in the next subsection. This very inaccuracy is part of the reason why the apparent “presentness” of information in perception needs to be explained as an informational construct, not as a feature of objective reality.

The idea of informational time marking is not novel. It has previously been used to account for perception of simultaneity in the face of different neural transmission latencies for tactile stimuli (Dennett & Kinsbourne, 1992), and for perceptual phenomena resulting from errors of synchronization for different features of stimuli with different processing latencies (Nishida & Johnston, 2002; see also Amano, Qi, Terada, & Nishida, 2016; Arstila, 2015; Derichs & Zimmermann, 2016; Moutoussis, 2012; Zeki, 2015). The most recent version of a time-marker hypothesis is a proposal by Herzog, Kammer, and Scharnowski (2016) that stimuli have duration labels attached to them. They argued that a stimulus with a duration of 50 ms does not result in a conscious percept of a 50-ms stimulus, but in a conscious percept with a duration label that indicates that it lasted for 50 ms. As Herzog et al. (2016) put it, “The stimulus is not perceived during the 50 ms when it is presented. The stimulus is even not perceived for a duration of 50 ms. Its duration is just encoded as a “number” (p. 5). According to Herzog et al. (2016), perceptual processing is nonconscious, and percepts emerge into consciousness with a latency of ~400 ms. Duration marking occurs during the initial 400 ms of nonconscious processing, so the conscious percept emerges with the duration marker attached to it.

The present proposal takes time marking further. The perceived present is itself a construction of time markers: not duration markers (though those are there as well), but time of occurrence markers. Thus, when a product of perception emerges, it emerges with a time of occurrence marker saying that it is occurring (in the outside world) now. All information in the perceived present is time marked as “present” and that, more than anything else, is what makes the information structure the perceived present. When information exits from the perceived present, if it is not lost, it is marked as “past” (by some amount of time). Given that information about “present” and past co-exist in the system at any given time, something is needed to differentiate them and to locate individual items in the stream of recent history as represented in perception and memory. Time marking has that function. Time marking as “present” solves two problems for defining and identifying a perceived present, the problem of variable processing latencies and the problem of varying temporal judgment thresholds. These will be addressed in turn.

### Time marking and variable processing latencies

There is now convergent evidence from multiple lines of research that the earliest emergence of a reportable visual percept is about 130–150 ms after stimulus onset (ASO; Anderson, Pederson, Sandberg, & Overgaard, 2016; Bagattini, Mazzi, & Savazzi, 2015; Koivisto & Revonsuo, 2010; Madec et al., 2016; Rossion & Caharel, 2011; Rutiku & Bachmann, 2017; Sandberg et al., 2013; Shafto & Pitts, 2015; Thorpe, Fize, & Marlot, 1996).^1^ That is already a problem because it implies that the perceived present is out of date by at least that amount of time. But many products of perceptual processing emerge with longer latencies than that. Some examples will be given to illustrate the range. In the case of face processing, Keyes, Brady, Reilly, and Foxe (2010) found differentiation between faces of friends and strangers around 250 ms ASO, but not earlier than that. Kiss and Eimer (2008) found differentiation between fearful and neutral faces around 400–600-ms ASO. Itier, Alain, Kovacevic, and McIntosh (2007) found differentiation between direct and averted gaze around 420–580 ms ASO. Construction of a perceptual map of the layout of the external world takes between 300 ms and 1,000 ms (Yoshimoto, Uchida-Ota, & Takeuchi, 2014; E. Zimmermann, Morrone, & Burr, 2014). Motion detection under conditions of noise can take as long as 3,000 ms (Burr & Santoro, 2001; Neri, Morrone, & Burr, 1998). In short, processing latencies for reportable visual percepts have a minimum of ~130 ms and vary on a time scale up to hundreds of milliseconds, if not more.

How can the products of processes with such variable latencies all be perceived as “present”? The answer is that whenever a product of perceptual processing emerges, it is entered into the perceived present and time marked as “present”. When a product of perceptual processing emerges, it does not emerge with an informational indication of its processing latency. That would result in a perceived present that was not a perceived present at all, but a perceived past with a disorganized mix of different time referents. One aim of perceptual processing is to construct a synchronized model of the world, a coherent and unified representation of stimuli in their proper temporal relations. Time marking newly emerged perceptual products as “present” contributes to that. The “present” marker is transitory. The fate of perceptual information and its time marking, and the transitoriness of the “present” marker, will be further elucidated in the section titled “Balancing Persistence Against Obsolescence”.

The previous paragraph is not meant to imply that differences in processing latencies are ignored in perception. There is evidence for attempts at compensation for differing processing latencies in the form of synchronization mechanisms. For example, there is evidence for cross-modal synchronization on time scales up to and even beyond 200 ms (Chen & Vroomen, 2013; Diederich & Colonius, 2015; Dixon & Spitz, 1980; Donohue, Woldorff, & Mitroff, 2010; Eg & Behne, 2015; Freeman et al., 2013; Love, Petrini, Cheng, & Pollick, 2013; Mégevand, Molholm, Nayak, & Foxe, 2013; Petrini et al., 2009; Van Wassenhove, Grant, & Poeppel, 2007). With repeated sequences of audiovisual stimuli with fixed delays between the event in one modality and that in the other, the brain gradually recalibrates so that the two events are perceived as simultaneous, and this too can happen on a time scale of 200 ms or more (Chen & Vroomen, 2013; Fujisaki, Shimojo, Kashino, & Nishida, 2004; Heron, Roach, Whitaker, & Hanson, 2010; Parsons, Novich, & Eagleman, 2013; Roach, Heron, Whitaker, & McGraw, 2011; Rohde, Greiner, & Ernst, 2014; Vroomen, Keetels, de Gelder, & Bertelson, 2004). Those processes, however, do not generate a “present” time marker, but only markers that identify two events as simultaneous. They could be identified as simultaneous and as occurring in the past. Time marking as “present”, therefore, is different from time marking as “simultaneous”.

Synchronization is not necessarily either accurate or universal in perception, and substantial perceptual asynchrony may go uncorrected (Arnold, Clifford, & Wenderoth, 2001; Dixon & Spitz, 1980; Durgin & Sternberg, 2002; Freeman et al., 2013; Halliday & Mingay, 1964; Ipser et al., 2017; Vroomen & Keetels, 2010). But, arguably, the very imperfections in synchronization support the “present” marking hypothesis. Marking all products of perceptual processing as “present” when they emerge, regardless of errors in or absence of synchronisation, smooths over temporal incoherence in perceptual information. Thus, time marking as present gives temporal coherence to sets of perceptual information that are generated with different latencies, just as integration of features gives spatial coherence to an individuated perceptual object.

### Different kinds of time markers: The past within the present

It might be thought that the time scale of the perceived present corresponds to the minimum temporal detection or discrimination threshold in perception: that the minimum time difference at which one stimulus can be perceived as occurring before another marks the time scale of the perceived present. In this section, it will be argued that this is not correct on the grounds that there are different kinds of time markers.

There have been many studies of minimum thresholds for judgment that two stimuli are temporally differentiated. Kinds of judgment include judgment that two stimuli were or were not simultaneous (nonsimultaneity judgment), judgment that there was or was not a temporal gap or discontinuity in a stimulus (gap judgment), and judgment that two stimuli occurred in a particular temporal order (temporal order judgment; TOJ). The collective term “temporal judgment threshold” (TJT) will be used for these, encompassing both detection and discrimination judgments.

A recent survey (White, 2018)^2^ has shown a great range of TJTs in studies of within-modality stimuli in the visual, auditory, and somatosensory modalities. Reported TJTs range from less than 1 ms (e.g., Henning & Gaskell, 1981; Zera & Green, 1993) to more than 100 ms (e.g., Fink, Ulbrich, Churan, & Wittmann, 2006; Marks et al., 1982). Many more thresholds fall between those extremes, and there is no obvious peak in the distribution. It is likely that the shortest TJTs do not indicate true temporal differentiation because there is evidence that judgments were made using nontemporal cues. To illustrate, Henning and Gaskell (1981) found a region of accurate TOJ for brief auditory stimuli with a gap of around 0.2 ms. However, the judgment was based on resemblance to different allophones of a consonant, not on detection of temporal order. What, then, is the shortest TJT at which there is compelling evidence for genuine temporal differentiation in perception? White (2018) concluded that such evidence could be found in studies reporting thresholds around 18–20 ms (e.g., Brown & Sainsbury, 2000; Craig & Baihua, 1990; Eimer & Grubert, 2015; Fink, Churan, & Wittmann, 2005; Hirsh & Sherrick, 1961; Nicholls, 1994; Stevens & Weaver, 2005; Tadin, Lappin, Blake, & Glasser, 2010). The true minimum could still be less than that, but evidence for that possibility is not compelling at present. It could be argued, therefore, that the perceived present has a minimum temporal resolution of ~20 ms. This is not meant to imply that the perceived present proceeds in units of 20 ms, or that the perceived present comprises frames of perceptual information updated at intervals of 20 ms. It means only that stimuli have to be separated by ~20 ms before they are perceived as occurring at different times. This could be regarded as a fundamental level of temporal granularity in perceptual information, with two caveats: (i) that a shorter minimum is still a possibility, and (ii) that the minimum could differ between modalities.

That seems to imply that the time scale of, and the persistence of information in, the perceived present is 20 ms, if not less. Yet it is proposed here that the persistence or holding time of information in the perceived present can be as much as ~100 ms. How can those different values be reconciled? The explanation is that they are just different things.

There could be several kinds of time markers. For present purposes it suffices to consider three: ordinal time markers, duration time markers, and time marking as “present”. Time marking as “present” is exclusive to the perceived present: when and only when a piece of information is in the perceived present, it is marked as “present”. This differentiates marking as “present” from all other kinds of time markers. A time marker of ordinality (including contemporaneity) can be attached to information about a stimulus at any point in processing, and it can be attached to that piece of information regardless of whether it is in the perceived present or not. If the piece of information leaves the perceived present and enters sensory memory or working memory, the ordinal time marker can still be attached to it there, but the marker of it as “present” is deleted. The same applies to a duration marker. If, as in the example from Herzog et al. (2016), a piece of information is labelled as a stimulus that lasted for 50 ms, that label can be attached to the piece of information before it enters the perceived present, while it is there, and after it has moved on to another store (if it does).

The possible minimum temporal resolution of 20 ms means just that one stimulus can be time marked as occurring after another if they are separated in time by at least 20 ms, and they cannot be so marked (or at least not with any guarantee of accuracy) if they are separated in time by less than that. Suppose two stimuli that are separated in time by 30 ms and are labelled as ordinally related. Information about the first stimulus enters the perceived present and is marked as “present”. After a short interval, information about the second stimulus enters the perceived present and is also marked as “present”. At that point, both stimuli are marked as “present”, but they are also marked as ordinally related in time: The first one is marked as occurring before the second. Thus, information about the first stimulus may be time marked as “present” because it is in the perceived present even though it is also perceived as preceding the second stimulus in time (and therefore, arguably, as in the past). Because the “present” marker and the ordinality marker are semantically distinct, there is no contradiction between them. In any case, a contradiction could only be registered if there was a process that took in both kinds of information and compared them, and any conflict such a process might detect would be very short lived. In the example considered here, the situation can persist only for up to ~70 ms. After that point, the first stimulus is no longer in the perceived present. If it survives (for example, in sensory memory), then it may still be labelled as ordinally related to the second stimulus, but it is no longer labelled as “present”. If two stimuli are perceived as more than 100 ms apart, then information about the two will not be in the perceived present at the same time, because the information about the earlier one will have gone from the perceived present before information about the later one has been entered into it. In such a case, the possibility of detecting a contradiction would never arise.

In short, the perceived present comprises all and only information time marked as “present”, and each item of information may have other time markers for ordinality and duration. The minimum TJT threshold merely places limits on labelling with temporal ordinality markers, and those labels are independent of the fact that the information is, at a given moment, also labelled as “present”.

## The perceived present as a holding area for perceptual information

### Perceptual processes and products

The perceived present is a set of products of perceptual (and other) processes. But what is the difference between a process and a product? In an information-processing model, a process is an operation that transforms information. The product is then the transformed information (White, 1988). The product is, in principle, accessible to other processes, whereas the process that generated it is not (Fodor, 1983; Scholl & Tremoulet, 2000). How does that apply to the perceived present? The problem is that information exists at all stages of perceptual processing. Even at the earliest low-level processing, with local processing of edge information, angle information, low-level motion information, and colour information (Holcombe, 2009), information is there, being operated on. The information that is there may be local and not integrated into coherent structures such as perceptual objects, but that does not alter the fact that information is as much there at that early stage as it is in any subsequent processing; and, once there, it can move on to subsequent processes. It could be argued, therefore, that products are being generated all through perceptual processing, not just at the end of it. That being so, what is the difference between information in the perceived present and all the information in perceptual processing prior to and leading up to the perceived present?

The answer to be proposed to that question starts to take us in the direction of elucidating the function of the perceived present. If it is there, it has a job to do, and its characteristics and features relate to the job that it does. It is proposed that the perceived present is a holding area for perceptual information. That is, what differentiates information in the perceived present from information at earlier stages of perceptual processing is that it is held as it is for a short period of time; that operations that transform it have ceased, at least temporarily. Thus, the perceived present is a body of information that is organized, synchronized, integrated, and marked with indicators of temporality, and it is neurologically active, but not at the moment being transformed in any way: It is just held as it is for some minimum amount of time. The perceived present is, in effect, a very short-term and very large capacity store or buffer for perceptual information.^3^ It is the holding of information that makes that information a product. Information does not naturally persist as physical objects do: It has to be made to do so by persistence or recurrence of neural activation. The perceived present is the enforced persistence of perceptual information. The distinction between process and product, then, is that process involves active construction or transformation or use of information, whereas product involves information being held as it is for some minimum amount of time.

The usual function of holding information in a store is availability to further processing. This has been proposed, for example, as part of global workspace hypotheses (Baars, 1988, 1997, 2002; Baars & Franklin, 2003; Dehaene, 2014; Dehaene, Charles, King, & Marti, 2014; Dehaene & Naccache, 2001). The term “global workspace” (Baars, 1988) or “global neuronal workspace” (Dehaene & Naccache, 2001) refers to a kind of central information exchange in the brain, subject to the limited capacity of working memory, but serving as a hub for the flow of information to or between specialized processors. The perceived present serves a similar function: There is no point in holding information if it is not to be potentially available to subsequent processing (Cleeremans, 2011; Farah, 1984; Fazekas & Overgaard, 2018). The kind of processing to which information in the perceived present might be available is a matter of conjecture. Global workspace hypotheses generally suppose that information in working memory is available to limited capacity higher cognitive processes (Baars, 2002; Dehaene & Naccache, 2001). The perceived present has a much higher capacity than the hypothesized global workspace and a much shorter duration, as will be shown in the section titled “Evidence for the Holding Area Proposal”. This indicates that information in it is likely to be used by rapid, high capacity automatic processes. One possibility is that there may be specialized “read-off” mechanisms that selectively access particular kinds of information and generate a judgment that requires minimal further processing. One possible example, exploiting the high capacity of the perceived present, would be a sample-size estimate, where a rapid scan of items simultaneously in the perceived present would automatically generate a quick and rough estimate of numerosity (Dehaene, Spelke, Pinel, Stanescu, & Tsivkin, 1999; Pica, Lemer, Izard, & Dehaene, 2004). Another possibility is transformation of information into other representational formats, such as linguistic format (e.g., for describing tastes, musical sounds, etc.). It is likely that much of the information in the perceived present is lost without entering further stores or processing, because of processing bottlenecks (Jacob et al., 2013; Öğmen, Ekiz, Huynh, Bedell, & Tripathy, 2013; Sperling, 1960) or interference from subsequent information input. Greene (2016) commented that the results of a study by Keysers, Xiao, Földiák, and Perrett (2001) suggested that “the information provided by a brief display can be compartmentalized and buffered against interference from information that follows as soon as 14 ms later” (p. 222). The perceived present could have just such a function. Availability allows selectivity: Some information is selectively attended to and further processed or stored, in accordance with prevailing priorities. By “stored” I mean that it crosses a bottleneck to further stores where it can be retained for longer, such as sensory memory and working memory. Having much information available increases the likelihood that what is needed can be found there.

### Balancing persistence against obsolescence

The effective minimum time that information spends in the holding area would be the minimum required for availability to further processing for that information to be significantly enhanced. This has to be balanced against updating of information: Information in the holding area that is not updated is liable to become obsolete, and there is then a need for it to be modified or replaced with the most recent available information. New products of perceptual processing are being generated all the time and enter the perceived present, potentially becoming confounded with more out-of-date information that is already there. If a piece of information becomes out of date due to some change in stimulus information, then there may be a need to remove it to minimize conflation or blurring with the new input. In that case, updating or overwriting may take priority over persistence.

In that respect, a parallel can be taken with visible persistence. Visible persistence refers to the persistence of surface visual features of a stimulus at retinal and cortical levels beyond termination of the stimulus (Breitmeyer et al., 1982; Coltheart, 1980; Di Lollo, 1977, 1980; Di Lollo & Bischoff, 1995). For stimuli with durations exceeding 200 ms, visible persistence can have a duration between 100 and 200 ms (Coltheart, 1980). With brief stimuli, however, the maximum duration of visible persistence is ~120 ms and the duration of visible persistence is inversely related to stimulus duration, so that it is zero with a stimulus of 120 ms duration (Breitmeyer et al., 1982; Coltheart, 1980; Di Lollo, 1977, 1980; Di Lollo & Bischoff, 1995; Efron, 1970; Greene & Visani, 2015; Sakitt, 1976; Yeonan-Kim & Francis, 2019). Why does the duration of visible persistence vary, and, in particular, why is it inversely related to stimulus duration?

It has been argued that visible persistence serves the function of maximizing the time available for visual analysis of the stimulus (Farrell, 1984). If a stimulus is very brief, say a few ms, then persistence of low-level analysis of the stimulus facilitates further (high level) analysis of it. The drawback is that, particularly when perceiving moving objects, visible persistence would result in motion smear. Farrell (1984) pointed out that motion smear is smaller than would be expected given the duration of visible persistence. She argued that, with visible persistence of 100 ms, an object moving at 60 mph across the field of view would generate about 9 feet of motion smear, more than actually occurs (Bedell, Tong, & Aydin, 2010; Burr, 1980; Marinovic & Arnold, 2013). There is, therefore, a trade-off between maximizing time available for processing and minimizing smear. Farrell (1984) found that visible persistence was reduced for a given stimulus when other stimuli were presented close to it in space or time, supporting the hypothesis that the visual system adjusts the duration of visible persistence in accordance with processing priorities. Thus, the visual system is resolving a conflict between different functional priorities: preserving stimulus information for further analysis matters (Fazekas & Overgaard, 2018), but preventing interference between the stimulus in question and other stimuli also matters, so preservation of stimulus information is curtailed to avoid that kind of interference when it is functionally advantageous to do so.

That kind of conflict may be salient, and resolved in a flexible way, in the perceived present as well. That is, there may be a maximum duration for any given piece of information in the perceived present, but the actual duration could be shorter depending on changing priorities. In the case of the perceived present, the advantage of information persistence is increased availability to postperceptual processing, not facilitation of perceptual processing, because perceptual processing is (locally) complete when that piece of information enters the perceived present. That is one respect in which the perceived present can be distinguished from visible persistence. But the principle of flexibility in accommodating competing processing priorities is similar. The main function of the perceived present, then, is to maximize availability of large amounts of highly processed, recent information about the outside world to further processing and storage.

Could visible persistence be regarded as an example of the brief retention of information in the perceived present? In the extreme case, could the perceived present just be visible persistence (and equivalent phenomena in other modalities)? In support of this, the maximum duration of visible persistence and that proposed for the perceived present are similar. Also, on at least some occasions, a percept of visible persistence is reportable. An example, apparently known since the time of Aristotle, is the reportable percept of an arc of light that occurs when a stick with a burning ember at one end is rotated (Di Lollo & Bischoff, 1995). The example illustrates the problems caused by persistence, because whatever lies behind the persisting image of the ember from the observer’s point of view is temporarily obscured by it.

On the other hand, there are ways in which the perceived present can be distinguished from visible persistence. First, visible persistence is at least partly a phenomenon of retinal processing (Sakitt, 1976; Yeonan-Kim & Francis, 2019), which places it well before the perceived present in the stream of visual processing. Cortical mechanisms are also involved in visible persistence, but probably at an early stage of perceptual processing (Coltheart, 1980; Farrell, 1984; Yeonan-Kim & Francis, 2019). The perceived present, by contrast, has been shown earlier in this paper to include products emerging later in perceptual processing. Second, if the perceived present were just visible persistence, then there would be no information in the perceived present about stimuli for which there is no visible persistence. Visible persistence, as we have seen, is inversely related to stimulus duration, which would have the unlikely consequence that very brief stimuli with visible persistence of 100 ms would be represented in the perceived present, whereas longer stimuli with durations of ~100 ms and no visible persistence would not.

Third, and most important, Coltheart (1980) distinguished visible persistence from informational persistence, with visible persistence referring purely to visual features of stimuli and informational persistence referring to semantic features, as well as occurring on a longer time scale. Because the perceived present is a body of information incorporating semantic information as well as visual features, this would seem to separate visible persistence from the perceived present. Thus, the phenomenal experience of visible persistence of a stimulus would be a consequence in the perceived present of the mechanisms that generate visible persistence, which operate in retinal and early cortical processing. In general, however, persistence of information in the perceived present is not the same as visible persistence because of the incorporation of semantic information in what persists. Therefore, visible persistence may make a contribution to the contents of the perceived present, but the majority of the contents of the perceived present are generated in different ways and by different mechanisms.

Informational persistence occurs on the time scale of sensory memory, which in vision is up to ~1,000 ms (Coltheart, 1980; Demkiw & Michaels, 1976; Schill & Zetzsche, 1995; Sperling, 1960). This is far beyond the proposed time scale of the perceived present. Thus, although the perceived present and sensory memory both contain perceptual object representations with semantic content, they differ both in time scale and in capacity, with the perceived present having greater capacity and shorter time scale. Sensory memory is separate from the perceived present because, if sensory memory was just the perceived present with decay of information over 1,000 ms, then there would be substantial blurring of information in the perceived present with every change in information, whether that be because of stimulus motion or eye movements or new stimuli replacing old ones. Visual percepts have less blur than would be predicted on the basis of visible persistence and there is evidence for mechanisms of motion deblurring in perception (Bedell et al., 2010; Burr, 1980; Marinovic & Arnold, 2013; Scharnowski, Hermens, Kammer, Öğmen, & Herzog, 2007; Tong, Patel, & Bedell, 2005; Westerink & Teunissen, 1995). In that case, given that sensory memory has a far greater time span than visible persistence, the small amount of blurring that occurs is not compatible with sensory memory being identified with the perceived present, or being in the same representational space as the perceived present. The perceived present is, therefore, not to be identified with either visible persistence or sensory memory.

### The ~100 ms perceived present and variable processing latencies

In the present proposal, the maximum persistence of information in the perceived present is hypothesised to be ~100 ms. How can this short persistence can be reconciled with long and variable processing latencies? Two points need to be made.

One point is that ~100 ms is the maximum duration of information in the perceived present after the stimulus that gave rise to it has changed or terminated (temporarily ignoring processing latencies for the sake of simplicity). If a stimulus continues for, say, 500 ms without change, as in presentation of a photograph of a face, then information about the face can persist in the perceived present for 500 + 100 = 600 ms. In effect, it is updated as continuing to be the same for 500 ms, and then may persist without further updating for up to 100 ms. The word “updated” is important: What persists in the perceived present is only a representation of what is the case now (i.e. the fact that the stimulus is still there, for example 400 ms after its onset, with the same caveat about processing latencies). The history of the stimulus is represented elsewhere, such as in sensory memory or working memory, and such memorial representations may include information about stimulus duration. But they are representations of the past of the stimulus, not its present. The duration of updating of information about the stimulus is 500 ms, and 100 ms is the maximum duration of updated information (without further updating) in the perceived present. If a stimulus has a duration of 1 ms, then information about it can persist in the perceived present for up to 101 ms (approximately). These are maximum possibilities: Persistence of information may be less than the maximum, as discussed in the previous subsection.

The other point is that entry to and persistence of information in the perceived present happens when the informational product of a process emerges. Thus, if temporal integration of motion information continues for 3,000 ms before an informational product emerges (Burr & Santoro, 2001), that product enters the perceived present at that point and no earlier. This does not mean that the information is out of date by 3,000 ms. Temporal integration over 3,000 ms can only occur if the stimulus continues to be presented for at least that long: If the stimulus terminated after 2,000 ms, then no product of temporal integration would be generated, and motion would not be perceived. If information input to the temporal integration process terminated at exactly the time at which the product of the temporal integration process emerged, then that product would persist in the perceived present for no more than ~100 ms.

In summary, the duration of persistence of information in the perceived present is independent of the processing durations of perceptual processing prior to entry into the perceived present.

### Evidence for the holding area proposal

Is there any evidence for the proposal that the perceived present is a brief store of information? There is very little, perhaps because nobody has been looking for it, but a study by Jacob et al. (2013) is relevant. They presented a visual prime followed by another visual stimulus with an onset asynchrony (SOA) ranging from zero to 2,000 ms. The SOA is an indicator of the processing time available for the first stimulus before the second stimulus is presented. Participants carried out two reaction-time tasks—a priming task in which they identified the prime stimulus as one of two possibilities, and a comparison task in which they judged whether the second stimulus was same as or different from the first. The study yielded evidence for three stages of prime stimulus processing. In the first stage, priming effects increased rapidly to a maximum at SOA = 133 ms, replicating the results of an earlier study by Vorberg, Mattler, Heinecke, Schmidt, and Schwarbach (2003). The second and third stages showed peaks in the comparison task at SOA = 240 ms, declining to an asymptotic minimum at 720 ms, and at SOA = 1,200 ms, declining thereafter.

The first of these three stages is of the most interest here. Jacob et al. (2013) followed Vorberg et al. (2003) in interpreting it as showing accumulation of information in a visuosensory buffer. They also argued that it corresponded to visible persistence. They interpreted the second peak at SOA = 240 ms as indicating iconic informational persistence. Iconic memory is the original name for sensory memory in the visual modality, and they did also use the term “visuosensory” (p. 1115) as an alternative to “iconic”, so they were in effect arguing for the peak at 240 ms as indicating entry to the visual version of sensory memory. There are two problems with those interpretations. One is that visible persistence does not involve accumulation of information. It is the mere persistence of a visual trace, occurring in both retinal and early cortical processing, and decays over time. As we have seen, with brief stimuli, the maximum duration of visible persistence is ~120 ms and the duration of visible persistence is inversely related to stimulus duration, so that it is zero with a stimulus of 120 ms duration. The prime stimuli used by Jacob et al. (2013) had 13 ms duration, so visible persistence would be predicted to be about 110 ms. This would be predicted to yield a peak at SOA = 13 ms and a decline thereafter. That does not fit with accumulation to a peak at SOA = 133 ms.

The second problem concerns the relationship between visible persistence and sensory memory. Jacob et al. (2013) argued that the second stage of processing with a peak at SOA = 240 ms indicated entry to visual sensory memory. If that is the case, what enters sensory memory cannot be visible persistence because visible persistence has decayed completely by ~120 ms ASO.

The results of the study by Jacob et al. (2013) are better interpreted in terms of the perceived present. The early rise to a peak in the priming task at SOA = 133 ms does indeed indicate accumulation of information about the stimulus in a buffer, but that information is the accumulating contents of the perceived present, as products of high-level visual processing emerge. In the study by Jacob et al., this process terminated around 133 ms ASO because the stimuli were relatively simple, coloured, static geometric shapes and high-level processing was essentially complete by 133 ms ASO. Also, repeated presentation of the same stimuli would speed processing (Bachmann, 1989; Bachmann, Luiga, Põder, & Kalev, 2003). But if entry to sensory memory occurs at SOA = 240 ms, what is happening between SOA = 133 ms and SOA = 240 ms? One answer is that perceptual processing continues, but that would imply a continued accumulation of information and a later first peak. Another possibility is that the existing information is simply held, subject to a decay function, for ~100 ms, and then enters sensory memory. Under that interpretation, the results indicate a duration for the perceived present of ~100 ms. That is the empirical basis for the proposed maximum duration of information in the perceived present. Of course, this is only one finding and the duration may vary depending on numerous stimulus presentation factors: The proposed ~100 ms maximum is just a hypothesis, a target for experimental tests. Notably, there was a blank field between the first stimulus and the second, and the paucity of information in that might facilitate persistence of information about the first stimulus. Regardless of whether the first peak represents the time course of perceptual processing or visible persistence, if the second peak represents entry to sensory memory, then there is still an interval of ~100 ms between emergence of a perceptual object, which, as we have seen, occurs around 130–170 ms ASO, and entry to sensory memory. More information can accumulate during that time, as processes with longer latencies generate products, but the information that emerged after 130–170 ms must be retained (subject to however much decay occurs) in order to be entered into sensory memory. It must, therefore, be held in some sort of buffer for that amount of time. If it is, then that buffer is the perceived present.

In contrast to the priming task used by Jacob et al. (2013), Öğmen et al. (2013) used stimuli of multiple objects in motion and tested ability to report motion direction of an object cued at the moment of motion offset. They also found evidence for three information processing bottlenecks that appear to correspond to the three processing stages identified by Jacob et al. (2013), although their data did not permit latencies for the three bottlenecks to be assessed. They identified the second and third stages as sensory memory and visual short-term memory, corresponding to the identifications of the second and third stages by Jacob et al. (2013). They identified the first stage as stimulus encoding, however. This fits with the hypothesis that the first stage represents accumulation of processed information in a brief store. So, the first of the three stages found in both studies has properties that correspond to those hypothesized for the perceived present, brief duration, very high capacity, and temporal location prior to sensory memory. The evidence does not yet take us deeply into the nature and features of this brief store, but it is at least consistent with the present proposal.

## Other possible functions for the perceived present

There are several other possible functions for the perceived present, and this account is not meant to imply that none of them could be the case: A multifunctional perceived present is not unlikely. I shall briefly discuss three possibilities, beginning by largely rejecting the one that might seem most obvious.

### Representing the present

It certainly seems as though the perceived present represents the present moment, as it has the subjective quality of doing so: Perception never seems to be about the past. In the present proposal, however, that impression is a consequence of the fact that information in the perceived present is time marked as present, and that is not a valid guide to its actual temporal location. There are other reasons for thinking that representing the present is not the primary function of the perceived present.

It has already been shown that in vision, it takes a minimum of about ~130 ms for any integrated product of processing to emerge, and much perceptual information emerges with longer latencies than that. Even the minimum latency of ~130 ms is slow compared to some other processes operating on visual information. Gollisch and Meister (2010) have shown that much complex processing of visual input occurs at the retina, including detection of motion and texture, various kinds of adaptation, including pattern adaptation and contrast adaptation, and summation over multiple neural inputs. Retinal processing of moving object stimuli involves a form of anticipatory response that effectively compensates for the latency of physiological and neurophysiological processes in the retina (Berry, Brivanlou, Jordan, & Meister, 1999; Palmer, Marre, Berry, & Bialek, 2015). Low-level cortical visual processing takes only about 30 ms and is largely complete by 100 ms ASO (Holcombe, 2009). It has been proposed that the amygdala is involved in detection of threats, and that it receives information through a fast but crude subcortical route, as well as through a slower route via cortical processing (LeDoux, 1996; Luo, Holroyd, Jones, Hendler, & Blair, 2007; Morel, Beaucousin, Perrin, & George, 2012). Luo et al. (2007) found responsiveness to fearful faces only 20–30 ms ASO, and in fact they found response in the hypothalamus/thalamus area only 10–20 ms ASO.

Those findings show that if it really mattered that the perceived present occurred as quickly as possible, then it would be possible for one to occur with a latency much less than 130 ms. It would not be much like the perceived present that we actually have, but it could in principle provide gist representations that would specify enough information for urgent practical purposes. If there was a need for a perceived present to have the minimum possible latency, then there would be one with a latency much shorter than what appears to be the case. It is likely, therefore, that richness of information, comprehensiveness, perceptual object construction, featural and spatiotopic integration, and detail of analysis are priorities for the perceived present, more than speed of processing and response.

### A guide to action

It is reasonable to suppose that the perceived present is important for the guidance of action (Fazekas & Overgaard, 2018). If I want to pick up a pen from my desk, for example, it is important to have accurate information about the contours of the pen and its location in my visual environment, to guide an accurate reaching and grasping action. For that function, high spatial acuity, exact identification of the object and its features, and differentiation of it from the rest of the visual world would obviously confer practical advantages. Interacting with static features of the environment could, therefore, be facilitated by a perceived present with the properties proposed here. Problems arise, however, when interacting with moving objects. There are two problems: One is that the temporal resolution of information in the perceived present may not be capable of supporting accurate interventions on moving objects; the other is that the information in the perceived present is out of date.

On the temporal resolution problem, it has been shown that professional sportspeople can time their interception of projectiles such as cricket balls with a temporal error as small as 2–3 ms, and even nonprofessionals can achieve accuracy to within 5–6 ms (Brenner & Smeets, 2015; Brenner, van Dam, Berkhout, & Smeets, 2012; McLeod, McLaughlin, & Nimmo-Smith, 1985). If the minimum temporal resolution of information in the perceived present is ~20 ms, it would not seem to be capable of supporting such accurate timing of interceptions. Problems associated with motion blurring, as discussed earlier, would also limit accurate timing of interceptions.

The likely solution to the temporal resolution problem lies with the evidence for two neuroanatomical pathways for visual information processing, the dorsal and ventral pathways (Mishkin, Ungerleider, & Macko, 1983; Ungerleider & Mishkin, 1982). According to Goodale and Milner (1992) and Goodale (2014), the dorsal pathway is specialized for the guidance of interactions with objects and the ventral pathway is specialized for constructing a visual model of the world. Action can be guided by perceptual information processed through the dorsal pathway that seems not to enter the perceived present (Goodale, Milner, Jakobson, & Carey, 1991), and indeed reactive actions can occur on a shorter time scale than that needed to construct relevant content in the perceived present (Brenner & Smeets, 1997; Castiello, Paulignon, & Jeannerod, 1991; Prablanc & Martin, 1992). There is, therefore, a functional dissociation between the perceived present, at least in vision, and the control and visual guidance of actions. Goodale and Milner (1992) argued that the two pathways are not completely separate, and indeed it would make sense for some degree of functional integration between the two kinds of visual processing to occur (Breitmeyer, 2014). However, evidence shows that patients who have no apparent impairment in perceiving and recognizing a certain kind of object may be considerably impaired in their ability to act on it, even when it is stationary (e.g., Jakobson, Archibald, Carey, & Goodale, 1991). Conversely, a patient who is unable to recognize objects and their features may still be able to interact with them appropriately and accurately—for example, by producing appropriate gaps between fingers and thumb when asked to grasp the object (Goodale et al., 1991; Whitwell, Milner, Cavina-Pratesi, Byrne, & Goodale, 2013). This double dissociation indicates that the primary function of the perceived present is not the guidance of action (Goodale, 2014). It must be supposed that processing through the dorsal route has functional features that would support the accuracy in timing that has been observed.

On the second problem, Nijhawan (2008) calculated that, with a ballistic projectile moving at 90 mph, if we assume that it takes about 100 ms for a percept of the projectile to be constructed, the perceived location of the projectile lags about 13 feet behind its actual location at any moment (see also Land & Mcleod, 2000). Nijhawan (2008) proposed a way of compensating for this: The visual system uses the available information to construct an extrapolation of the ball’s trajectory that essentially predicts where it is at the present moment. Generalizing, the whole of the perceived present would be an informational extrapolation to the present: Unpredicted events are fitted in as quickly as possible. It can be argued that that extrapolation effectively compensates for the inaccuracy caused by the processing latency, to the extent that it accurately represents the next 100 ms of the projectile’s trajectory. Under that argument, the perceived present could be an extrapolation to the present that supports accurate timing of interceptions on moving objects by effectively predicting where they are in the present in the world outside the brain.

Unfortunately, that kind of extrapolation does not supply what is needed. The interception of moving objects requires extrapolation *beyond* the objective present, to the point in time when contact will occur or is predicted to occur. The times in the two extrapolations are different, though they converge as the moment of interception approaches. It has been found that contact times of moving objects that are 2,000 ms in the future can be predicted to an accuracy of 10 ms (Bootsma & Oudejans, 1993). That is a much longer extrapolation than would be involved in extrapolating to the present. Thus, when a cricket ball is bowled to a batsman, in the early stages of the ball’s flight, extrapolation to the present would place the ball some feet ahead of its location in the most recent available perceptual information but still some distance short of contact, but the perceptual system is also extrapolating a trajectory to the anticipated time and location of contact. Both extrapolations may be adjusted as the ball approaches and, as has already been said, they gradually converge on the time when the ball is contacted, where they coincide. But they are different. The hypothesis that extrapolation to the present may be involved in the construction of the perceived present is viable in its own right, but it does not serve the function of guiding the interception of moving objects.

### To keep everything from being present at once^4^

In the present account, the perceived present is located at or near the end of perceptual processing and prior to entry of information to sensory memory and/or working memory. Because all of the information in all of those processing stages is neurologically active, in a sense it is all there “now”. The main problem with that is that all of it covers or concerns a considerable span of time, not less than many seconds. If all of it was experienced as “now”, then there would be a huge conflation of information covering that long span of time. This will now be discussed.

On the input side, as we have seen, processing takes time, and the amount of time taken for perceptual products to be constructed varies over a substantial time scale, most occurring within a few hundred ms ASO, but some extending into the supra-second time scale (Burr & Santoro, 2001; Neri et al., 1998). The information that is in the system, in varying stages of construction, cannot all be designated as “now”—not just because there is so much of it, but because it reflects input occurring over that long time span. If we take vision as an example, even 100 ms of visible persistence is apparently not acceptable under some circumstances (Farrell, 1984): A few hundred milliseconds of visible persistence for all perceptual information would result in an amount of smearing that would render the visual world blurred to the point of incoherence and uselessness. This could be addressed by assigning information to separate buffers, the contents of which do not interfere with each other, and by allocating timing information. Thus, the perceived present could be part of that functional differentiation, a buffer of brief duration where information is designated as present, and separate from temporally prior information in perceptual processing.

On the other side of processing from the perceived present, there is rapid decay of information through sensory memory, on a time scale of about 1,000 ms (Demkiw & Michaels, 1976; Haber, 1983; Jacob et al., 2013; Schill & Zetzsche, 1995; Sligte et al., 2010; Sperling, 1960). It could be argued that the decay of information to an amount much smaller than that in the perceived present would minimize smear, but the problem is that the temporal extent of the information is much greater, 1,000 ms as opposed to approximately 100 ms maximum for the perceived present. That would result in a rapidly fading smear, but over a much greater temporal extent. It should be emphasized again that the smear would be due to information from different times being in the same representational space.

Some idea of the problem can be gained by studying cases of palinopsia. This has been defined as “the persistence or recurrence of visual images after the exciting stimulus object has been removed” (Bender, Feldman, & Sobin, 1968, p. 321). Gersztenkorn and Lee (2015) identified eight categories of palinopsia. The one most relevant for present purposes is visual trails (Horton & Trobe, 1999; Ihde-Scholl & Jefferson, 2001). The visual effect is not a smear or a streak but rather a series of discrete images that persist behind a percept of a moving object and gradually fade on a time scale of seconds. Two patients studied by Horton and Trobe (1999) reported seeing a stream of static images in the wake of a moving object that they were visually tracking. The visual trails interfered considerably with ongoing perception. When motion stopped, “the images collapsed into each other” (p. 530). Visual trailing was specific to perception of some (not all) moving objects. If it happened for all perceptual information, perception would be completely disrupted. Clearly, keeping everything from being present at once (at least on the subsecond time scale) is important, because the palinopsia cases illustrate the disabling effects of motion trails (driving a vehicle was impossible for the patients, for example; Horton & Trobe, 1999).

So, there is a need for a body of information that has the temporal specificity of the perceived present. It may indeed be that one of its functions is to prevent too much perceptual and memorial information from co-occurring in the same representational space. Whatever sensory memory might be, it is a separate buffer from the perceived present with different time markers.

## Other possible perceived presents

It is easy to imagine a perceived present quite different from the one we have. A perceived present that had properties similar to that of the hypothetical objective present would comprise a series of momentary snapshots on a minute time scale, lacking all historical information, and each obliterated as soon as the next was constructed. At the other end of the range of possibilities, consider the patient with akinetopsia described by Zihl, von Cramon, and Mai (1983). Her reports of her own experience indicated a visual world that proceeded in a series of static frames. For example, when pouring tea into a cup, “the fluid appeared to be frozen, like a glacier” (p. 315) and she could not judge when to stop pouring because she could not see the level of fluid in the cup rising towards the brim. She reported other experiences of a similar character. Spatial coherence was not lost, and her percepts included semantic information (she knew what a cup was, for example). After extensive testing, Zihl et al. concluded that the deficit was selective to movement vision. The patient retained information about the previous frame after it had been replaced by a new one, and the frames seemed to remain static for an appreciable fraction of a second, if not longer, rather than being replaced on the millisecond time scale. There seems to have been no blurring, as would happen if input information was smeared across the time span of a single long frame. It is as if a single moment was preserved, and all information between that and the next one to be preserved was lost, never part of the perceived present. There is no reason to think that the patient’s perceived present was directly affected; any effect on information in it would be a consequence of the motion perception deficit, and in any case would not generalize to other kinds of perception or other sensory modalities. The case is just being used here as a way of imagining different possible perceived presents.

Akinetopsia and the varieties of palinopsia discussed earlier illustrate the possibility that the perceived present could be very different from the one we actually have. The perceived present is as it is for functional reasons. The problems caused by the disorders help to elucidate the functional adaptation of the perceived present we have. It is not that there are no other options.

Although there is other perceptual information—in particular, information that guides action, such as predator avoidance, through the fast amygdala route, or projectile interception, through the dorsal route—it is the perceived present that seems to anchor us in, even to constitute, the present moment in the world beyond the senses. That can be explained in terms of time marking. Information in the perceived present is informationally marked as “present”. Even two stimuli that are perceived as nonsimultaneous can both be informationally marked as “present”; this is not incompatible with a separate set of markers indicating their temporal order. A time marker of “present” is a transient feature of a piece of perceptual information: If the present account is right, it will endure no longer than 100 ms and then change to a marker indicating some time in the past, unless the information is lost altogether.

The hypothesis that the most recent perceptually available information is used to construct a percept that is actually an extrapolation to the present state of the outside world was briefly discussed in the section titled “A Guide to Action”, and there is evidence to support it (Changizi, Hsieh, Nijhawan, Kanai, & Shimojo, 2008; Nijhawan, 1994, 2008; Nijhawan & Wu, 2009; Shi & Nijhawan, 2012). The hypothesis is controversial: Alternative explanations have been proposed for some of the findings claimed as supporting the extrapolation to the present hypothesis, and the extrapolation hypothesis does not seem to be able to explain some findings (Arstila, 2015; Briscoe, 2010; Eagleman & Sejnowski, 2007; Hubbard, 2014; Krekelberg & Lappe, 2001; Maus, Khurana, & Nijhawan, 2010; Purushothaman, Patel, Bedell, & Öğmen, 1998; Vaughn & Eagleman, 2013). Nevertheless, it remains possible that extrapolation plays an important part in constructing the information in the perceived present and compensating for the time delays that occur in doing so.

Whether or not that is the case, extrapolation to the present is mentioned here mainly to distinguish it from time marking. In the present proposal, marking information in the perceived present as “present” occurs regardless of whether or not that information is the product of an extrapolation process. Conversely, the hypothesis of extrapolation to the present does not require that extrapolated information be marked as present, and time marking does not appear to have been discussed in connection with extrapolation to the present. The two hypotheses sit comfortably together because it makes sense for information labelled as “present” to be an extrapolation to the likely present state of affairs, but they are not necessary bedfellows.

## Summary of proposed features of the perceived present

This section draws together some of the things that have been claimed about the perceived present in the foregoing account.

1. The perceived present is not a moment, but a functionally definable stage in the processing of perceptual information. Information enters that stage when perceptual processing of it has reached an end point. It is a set of informational products of perceptual processing, where “products” means that the informational products are held as they are for some amount of time.
2. Large information capacity. The perceived present is the earliest processing stage at which information is integrated into coherent objects and contexts. Information storage capacity, in terms of both number of items, complexity of features of perceptual objects, and integration of perceptual information into a coherent global representation (E. Zimmermann et al., 2014) is high in the perceived present. The information bottlenecks identified in the studies by Öğmen et al. (2013) and Jacob et al. (2013) indicate that subsequent processing stages, including sensory memory and working memory, are subject to progressive reduction in capacity. Capacity in working memory is usually defined in terms of number of discrete items (Cowan, 2001; Joseph, Teki, Kumar, Husain, & Griffiths, 2016; Schurgin, 2018), and some kinds of information may be more likely to enter working memory than others (Sachs, 1967), but capacity in the perceived present refers to the whole integrated collection of featural, object, and contextual information.
3. Subjective coherence and organization of information. The contents of the perceived present appear to cohere as an organized representation of what is out there, including such things as object individuation and binding, cross-modal synchronization and integration, and some kind of map of the perceived world (E. Zimmermann et al., 2014). The term “subjective” is used because there is probably some degree of local independence, with different pieces of information brought together and integrated when need be (e.g., Hogendoorn, Verstraten, & Johnston, 2010).
4. Capacity to represent information with temporal resolution on the millisecond time scale, down to at least 20 ms and possibly less.
5. Temporal integration. Specifically, the perceived present is not like a photograph in presenting an isolated moment: It captures some aspects of happening, such as object motion and ordinal temporal relations.
6. Holding information. The perceived present is a store of information with a duration that is variable up to a maximum of about 100 ms.
7. Incorporation of semantic information. Two examples are information about emotional expression in faces (e.g., Kiss & Eimer, 2008) and information about unobservable kinetic variables such as force, mass, and causality in perception of objects moving and interacting (White, 2009, 2012). The information in the perceived present clearly goes beyond superficial physical features.
8. Time marking. Information in the present is informationally marked as “now”, and other kinds of time markers, including ordinality, temporal interval, and duration, also occur.
9. Information in the perceived present may be accessed by other processes (e.g. read-off mechanisms, automatic perceptual learning processes) and may be transferred to sensory memory or working memory, but is subject to rapid decay from its initial high capacity to the initial capacity of sensory memory.
10. Continuity in the waking state. The perceived present goes on all the time, at least in the waking state.

The perceived present is, perhaps, the ultimate illusion—the use of informational markers to generate the impression that we perceive what is going on now, when, in fact, perceptual processing latencies entail that this is not the case.

This summary helps to show how the perceived present differs from visible persistence, which is a local phenomenon of early visual processing prior to incorporation of semantic information (Coltheart, 1980). The 10 features listed above are not necessary, and it is possible to imagine a perceived present different in almost every respect: one that represented purely superficial features of objects, such as shape and luminance, without semantic information, or one that had a longer holding time, as in akinetopsia. The features in the list certainly are not features of the hypothetical present moment in physics, which has a temporal resolution on the Planck time scale, lacks semantic information, and has coherence that is bestowed on it by the laws of physics, not by information processing. The particular features of the perceived present must speak to the functions that it has: different functions would require or favour different features.

## Conclusion

The perceived present is an important but neglected research topic. Those who research perceptual processing are concerned with things that go on before information enters the perceived present; those who research memory, from sensory memory on, are concerned with what happens to information after it leaves the perceived present. Those who research topics related to consciousness such as the time to emergence of conscious percepts are concerned with a problem that is, at most, only a part of what matters about the perceived present. The perceived present itself, as an information structure, has been neglected. By showing that it has informational features that do not correspond to features of the hypothetical present moment of physics, and by considering hypotheses about its functional significance, I hope to have shown that studying the perceived present is important to a full understanding of the long and complex stream of perceptual and memorial processing.

Something is registered as being in the past, not because it is, but because it is time marked as being in the past. It must be the same for the present: that is how past and present are differentiated in perception and memory. Time is never directly experienced. All experience of time is in the form of time markers that temporally differentiate perceptual information in various ways.

There is clearly a need for research focussed on the perceived present and its properties. Those who have postulated frames of conscious perception are among the few who have attempted that: A frame has the explanatory advantage that information in a given frame is held without change for the time span of the frame. However, the evidence for frames is not compelling (White, 2018), and there is much about the process of frame construction that is not understood. Piecemeal updating of the perceived present is a viable alternative, but there is still a need for a mechanism for holding information for the duration of the perceived present. At least the debate between frames and piecemeal updating is broaching the problem of the perceived present from one direction, and the studies by Jacob et al. (2013) and Öğmen et al. (2013) have made a start on investigating the properties of the perceived present by looking at build-up and decay of information on short time scales. Hopefully, these lines of research can be further developed into a more complete body of research on the problem.

## Abbreviations

ASO: after stimulus onset
TOJ: temporal order judgment
TJT: temporal judgment threshold
SOA: stimulus onset asynchrony.

## Footnotes

1 This conflicts with the evidence for latency to conscious perception of ~400 ms reported by Herzog et al. (2016). At present, it is not clear how this conflict can be resolved (see, e.g., Koivisto & Revonsuo, 2010; Rutiku & Bachmann, 2017).

2 Copies of this paper are available from the author on request.

3 Interestingly, a buffer on the time scale of temporal integration in perceptual processing has also been proposed by Rüter, Marcille, Sprekeler, Gerstner, and Herzog (2012) as a component in a two-stage model of rapid decision-making. The duration of information storage in the buffer was not specified by the authors, but would have to be on the millisecond time scale. In the present proposal, there is one buffer for the entire perceived present, but the possibility of multiple buffers for specific processing functions should not be discounted.

4 The section heading is adapted from the statement “time . . . is what keeps everything from happening at once”, sometimes attributed to the physicist John Wheeler, but originating in a novella by Ray Cummings (1922).
